# Patterned Polyvinyl Alcohol Hydrogel Dressings with Stem Cells Seeded for Wound Healing

**DOI:** 10.3390/polym11010171

**Published:** 2019-01-18

**Authors:** Tianlin Gao, Menghui Jiang, Xiaoqian Liu, Guoju You, Wenyu Wang, Zhaohui Sun, Aiguo Ma, Jie Chen

**Affiliations:** 1 The College of Medical, Qingdao University, Qingdao 266021, China; gaotl@qdu.edu.cn (T.G.); jiangmenghui@qdu.edu.cn (M.J.); 2014210314@qdu.edu.cn (X.L.); 2014210316@qdu.edu.cn (G.Y.); 2015210300@qdu.edu.cn (W.W.); 2015210291@qdu.edu.cn (Z.S.); 2 Key Laboratory of Polymer Ecomaterials, Changchun Institute of Applied Chemistry, Chinese Academy of Sciences, Changchun 130022, China

**Keywords:** PVA hydrogel-based dressing, photo-immobilization, stem cell, bioactive factors, wound healing

## Abstract

Polyvinyl alcohol (PVA) hydrogel and stem cell therapy have been widely used in wound healing. However, the lack of bioactivity for PVA and security of stem therapy limited their application. In this study, an adipose-derived stem cells (ADSCs)-seeded PVA dressing (ADSCs/PVA) was prepared for wound healing. One side of the PVA dressing was modified with photo-reactive gelatin (Az-Gel) via ultraviolet (UV) irradiation (Az-Gel@PVA), and thus ADSCs could adhere, proliferate on the PVA dressings and keep the other side of the dressings without adhering to the wound. The structure and mechanics of Az-Gel@PVA were determined by scanning electron microscopy (SEM) and material testing instruments. Then, the adhesion and proliferation of ADSCs were observed via cell counts and live-dead staining. Finally, in vitro and in vivo experiments were utilized to confirm the effect of ADSCs/PVA dressing for wound healing. The results showed that Az-Gel was immobilized on the PVA and showed little effect on the mechanical properties of PVA hydrogels. The surface-modified PVA could facilitate ADSCs adhesion and proliferation. Protein released tests indicated that the bioactive factors secreted from ADSCs could penetrated to the wound. Finally, in vitro and in vivo experiments both suggested the ADSCs/PVA could promote the wound healing via secreting bioactive factors from ADSCs. It was speculated that the ADSCs/PVA dressing could not only promote the wound healing, but also provide a new way for the safe application of stem cells, which would be of great potential for skin tissue engineering.

## 1. Introduction

The ideal wound dressing maintains a moist environment around the wound, absorbs the exudates from its surface, and is qualified with biological functionality [1]. Hydrogels have been widely used as ideal tissue-engineering scaffolds for their high water content and structural similarity to the native extracellular matrix (ECM) [2,3,4]. Polyvinyl alcohol (PVA) hydrogels have been studied for biomedical applications including soft contact lenses, cartilage implants, drug-delivery matrices, temporary skin covers or burn dressings and artificial organs, because of their inherent non-toxicity, non-carcinogenicity, good biocompatibility and desirable physical properties such as rubbery or elastic nature and high degree of swelling in aqueous solutions [5,6]. When applied as wound dressings, the PVA hydrogels could maintain the moist environment and absorb the exudates from the wound surface [7]. Furthermore, as the non-adhesive property of the PVA hydrogel dressings, the dressings could be easily separated from the wound in the process of changing fresh dressings without new wound injury. However, a lack of biological activities seriously hindered the application of PVA hydrogels in wound healing [8].

Multipotent mesenchymal stem cells have been widely used in tissue engineering for their potential to differentiate into multiple mesodermal tissue types. The stem cell therapy, employed with different cell types and methods, has been widely studied in wound healing [9,10,11]. Adipose-derived stem cells (ADSCs) have attracted much attention as they can be relatively easy isolated and cultured. Lots of studies showed that ADSCs can not only possess the ability to differentiate into a variety of cell phenotypes, like osteocytes, smooth muscle cells and endothelial cells, but also secrete potent combinations of bioactive factors like vascular endothelial growth factor (VEGF), insulin-like growth factor-1 (IGF-1), and basic fibroblast growth factor (bFGF) related to paracrine actions [12,13]. These factors both benefit for wound healing [14,15]. However, it was found that eight of nine late-passage human embryonic stem cell (hESC) lines had one or more genomic alterations commonly observed in human cancers [16,17]. The secure application of stem cells in tissue engineering should be seriously taken into account. We speculated that the stem cells, separated by hydrogel dressings from the wound, could not only avoid direct contact with wound to reduce cancer risk, but also secrete bioactive factors through pore of hydrogel dressings onto the wound to accelerated tissue repair.

However, the inherent property of PVA hydrogel is not conducive to the stem cell adhesion, so that the stem cells could not adhere, proliferate on the hydrogel and secret enough bioactive factors. Gelatin, obtained by the denaturation of collagen, was selected as the biological polymer for incorporation and modification due to its low immunogenicity, biodegradability, and biocompatibility [18]. Physically blending in synthetic hydrogels is a simple but effective method to improve the hydrogels bioactivity. However, the proteins leach from the hydrogels typically in a relatively short incubation time [19]. In addition, it was reported that incorporating a high concentration of gelatin into hydrogels affects their diffraction, mechanical properties, crystallinity and swelling behaviors [20]. Furthermore, after the blending of gelatin, the dressings could also be conducive to cell adhesion of the wound and adhere with the wound, which is not convenient for change of new dressings. Our co-worker reported that a photo-reactive gelatin (Az-Gel), which could be immobilized on various matrices by UV-irradiated, was synthesized to either improve the matrices biocompatibility or immobilized bio-signal molecules onto them [21,22]. Using photo-reactive gelatin to improve the surface properties of PVA hydrogel dressings could not only make the surface of the dressings conducive to the stem cell adhesion, proliferation and secrete active factors, but also make the bottom of the dressings not stick to the wound.

**Scheme 1:**
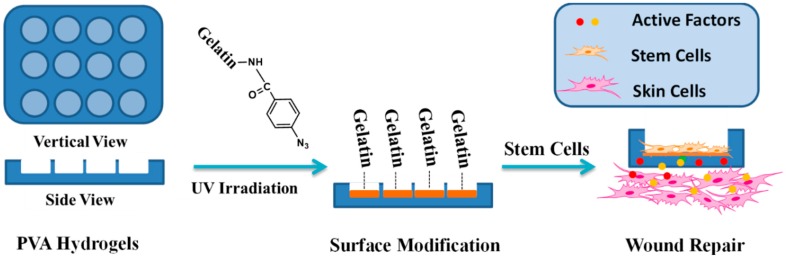
Schematic illustration of patterned polyvinyl alcohol (PVA) dressings seeded with adipose-derived stem cells (ADSCs) for wound healing.

In this study, we report a biological safe PVA hydrogel dressing with a groove structure in which mouse ADSCs were seeded so that the cells could not proliferate out of the groove. The surface of the groove was photo-immobilized with Az-Gel (Az-Gel@PVA) to facilitate the adhesion and proliferation of ADSCs. After the ADSCs seeded in the groove, the cell-seeded dressings (ADSCs/Az-Gel@PVA) were applied in wound healing and the active factors secreted from ADSCs penetrated through the dressings and accelerate the wound healing (Scheme 1).

## 2. Materials and Methods

### 2.1. Fabrication of Patterned Polyvinyl Alcohol (PVA) Dressings

**Figure 1:**
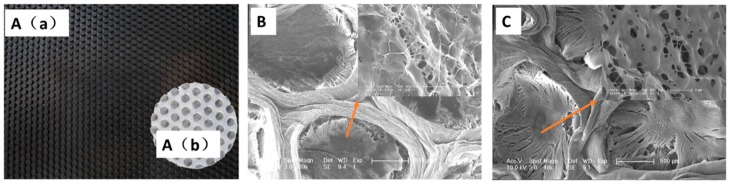
Optical images (**A**(a) and **A**(b)) and scanning electron microscope (SEM) images (**B** and **C**) of patterned PVA dressings: the honeycomb mould (**A**(a)), the patterned PVA dressings (**A**(b)), Az-Gel@PVA dressings (**B**) and PVA dressings (**C**).

The PVA (Alcoholysis 99.8–100% (mol/mol), Aladdin, Shanghai, China) dressings were fabricated by a frozen-thawed method. Briefly, aqueous solutions of 12% (*w*/*v*) PVA was prepared by dissolving preweighted quantities of PVA in distilled water and heating at 90 °C for 6 h. The PVA solution was then poured into a plate with a honeycomb mould (Figure 1A(a)) at the bottom. Then the plate was frozen at −20 °C for 12 h and slowly thawed for another 12 h. This physical crosslinking procedure was repeated for 3 times.

### 2.2. Surface Modification via Photo-Reactive Gelatin (Az-Gel)

The photo-immobilization of Az-Gel was performed as followed. Az-Gel was synthesized according to our previous study [22]. Az-Gel was dissolved in distilled water and the aqueous solution (1 mg/mL) was cast on the surface of the grooves and air-dried at room temperature. The dressings was then UV-irradiated using a UV lamp (CL-1000 Ultraviolet Crosslinker, UVP, Upland, CA, USA) from a distance of 5 cm for 20 s (16 mW/cm^2^) and washed repeatedly with warm distilled water (37 °C) to remove the unfixed Az-Gel. After being sterilized with 75% ethanol, the dressings were incubated in sterile phosphate buffered saline (PBS) solution.

### 2.3. Structure and Mechanical Properties of PVA and Az-Gel@PVA Dressings

A scanning electron microscope (SEM) (XL30 ESEM-FEG, FEI, Hillsboro, OR, USA) was used to observe the structure of the Az-Gel@PVA dressings. Dressing samples were freeze-dried, mounted on metal stubs with double sided tape, coated with chromium and observed by SEM with accelerating voltage 10 kV.

The tensile strength and breaking elongation of scaffolds were determined using a material testing instrument (Instron-1211, Instron, Boston, MA, USA). The scaffolds were cut into a specific dog bone shape (10 cm long, 2 cm wide at the ends and 1 cm wide in the middle, 1mm thick). The mechanical analysis was performed at a stretching rate of 10 mm/min with preload of 0.5 N to determine the maximum load for each matrix [23].

### 2.4. Adipose-Derived Stem Cells (ADSCs) Adhesion and Proliferation on the Az-Gel@PVA Dressing

ADSCs were isolated from adipose tissues obtained from BALB/c mouse using an established protocol as before [24]. Briefly, the adipose tissue were extracted and washed with phosphate buffered saline (PBS) on a separating sieve. After treated with collagenase type I (Gibco, New York, NY, USA), cells were collected by centrifugation and plated in tissue culture flasks along with Dulbecco’s Modified Eagle Medium (DMEM, Gibco, New York, NY, USA) supplemented with 10% fetal bovine serum (Gibco, New York, NY, USA), 100 U/mL penicillin and 100 U/mL streptomycin (Sigma, San Francisco, CA, USA). After the cells were attached, the medium was replaced with fresh medium. ADSCs of passages 2 or 3 were used for the experiments in this study.

PVA dressings were prepared under sterile conditions, and then soaked in the culture medium overnight; 400 μL ADSCs suspension was seeded onto the dressings in 48-well plates at the concentration of 1 × 10^5^ cells/mL. After 1, 4, 24 and 48 h culture, live-dead assay (Sigma, San Francisco, CA, USA) was done to determine the adhesion of cells on the surface of the grooves. In brief, the dressings were washed with PBS then stained with 4 mmol/mL propidium iodide (PI, Sigma, San Francisco, CA, USA) and 2 mmol/mL Calcein-AM (Cal-AM, Sigma, San Francisco, CA, USA). After 10 min incubation with the stains at 37 °C, the dressings were washed again with PBS and images were captured using fluorescent microscope (TE2000-u, Nikon, Tokyo, Japan). Meanwhile, the cell proliferations after different times (1 and 4 days) were determined via cell count. The cell counting method was as follows. The cells in each plate are digested by 0.25% trypsin (Sigma, San Francisco, CA, USA) and collected after centrifuging 5 min at 1000 rpm. Then the cells were re-suspended in 1 mL medium and a drop of the cell suspension was added to the hemocytometer (MARIENFELD, Germany) for cell counting.

### 2.5. Total Protein Release

As is known, the bioactive factors secreted by ADSCs contains many kinds of proteins. To crudely reflect the secreted bioactive factors released from the ADSCs/Az-Gel@PVA dressing, ADSCs with different concentrations were seeded on the Az-Gel@PVA dressings and incubated in serum-free medium for 48 h. Then the total protein release into the medium was tested via Protein Assay Kit (BCA, Solarbio, Beijing, China) at 562 nm on a multifunctional microplate scanner (Tecan Infinite M200, Mannedorf, Switzerland). The bFGF release was also specifically assessed via enzyme-linked immunosorbent assay (ELISA) kit to further determine the bioactive factors from the ADSCs/Az-Gel@PVA dressing. Briefly, the medium was centrifuged at 4000 rpm at 4 °C for 10 min, then the supernatant was collected and assessed using an ELISA kit according to the manufacturer’s instructions (R&D, Minneapolis Sue, MN, USA). After incubated with biotin-conjugated anticytokines primary antibodies, washed and incubation with HRP-conjugated streptavidin. The color was developed using the enzymatic substrate *o*-phenylenediamine and the optical density values of absorbance were read on the multifunctional microplate scanner.

### 2.6. NIH-3T3 Cell Proliferation and Scratch Experiment

The mouse fibroblast NIH-3T3 cell line was chosen as the model cell and cell proliferation was performed as follows. Briefly, NIH-3T3 cells were seeded in 12-well at 2 × 10^4^ cells/well. After overnight incubation, the PVA dressing and ADSCs seeded dressings were placed on the NIH-3T3 cells covering the plate. The NIH-3T3 cell proliferation was analysed by Cell Counting Kit-8 (CCK-8, Dojindo, Kumamoto, Japan) after removing the dressings. The absorbance values at 450 and 600 nm were measured using a multifunctional microplate scanner after 2 h of incubation.

Then, the cell scratch experiment was performed to observe the migration of cells [25]. Briefly, NIH-3T3 cells were cultured in 12-well plates at 1 × 10^5^ cells/well. When the cells covered the bottom of the well, a scratch was made with a sterilized P1000 pipette tip (a width of 0.4–0.5 mm). After cleaning the stripped cells with PBS, the NIH-3T3 cells continued to be cultured for 24 h, covered with ADSCs-seeded PVA dressings. Then, the NIH-3T3 cells were fixed in 4% paraformaldehyde and stained with 0.4% Trypan Blue (Gibco, New York, NY, USA). The scratches were analyzed with a microscope and measured with “NIH ImageJ” software.

### 2.7. Animal Experiment

Eight-week old healthy male Sprague–Dawley Rats were used for experiments. Before starting the surgery, rabbits were anaesthetized by giving intravenous injection with 7% chloral hydrate (5 mL/kg). Thereafter, an electric shaver was used to remove hair from the dorsal area and skin was sterilized using 75% ethanol. According to the principle that the least number of animals were used, four full thickness square defects were created (1 cm × 1 cm) on each rat. Among the four defects, two defects were covered with ADSCs/Az-Gel@PVA dressings, while another two defects were covered with Az-Gel@PVA dressings as control. Also, the dressings were replaced every two days. The rats were housed individually in the cages and the wound healing was observed after 2 weeks. The closure of wound was used to reflect the wound healing. In brief, the wounds were captured at 0, 1 and 2 weeks post-surgery using a digital camera (Canon, Tokyo, Japan). The wound area was detected via image analysis software (ImageJ). The degree of wound closure was according to the formula:

Wound closure (%) = (A_0_ − A_t_)/A_0_ × 100, (1)

where A_0_ is the initial wound area and A_t_ is the wound area at each time point.

### 2.8. Statistical Analysis.

The data presented are the mean ± standard deviation (S.D.). Independent and replicated experiments were used to analyze the statistical variability of the data using one-way analysis of variance (ANOVA). Differences were set at *P*-values less than 0.05 (*P* < 0.05).

## 3. Results

### 3.1. Patterned PVA Dressings with Surface Modification

The patterned PVA dressings was fabricated using a honeycomb mould (Figure 1A(a)). After repeating three times of frozen-thawed process, the patterned PVA dressings were full of circular grooves (Figure 1A(b)). SEM images showed that unmodified PVA dressings kept a typical structure of the PVA hydrogel with pores through each other (Figure 1C). However, there were many filaments found interlaced between the skeletal structures on the surface of the grooves modified with Az-Gel (Figure 1B), whereas no such filaments were observed on the PVA hydrogels without Az-Gel modification (Figure 1C).

### 3.2. Mechanical Properties of Az-Gel@PVA Dressings

**Figure 2:**
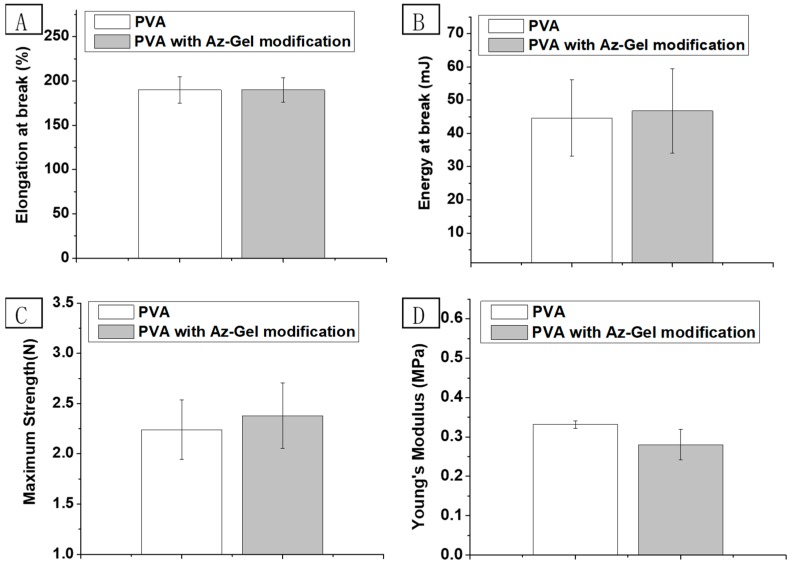
Elongation (**A**), energy at break (**B**) maximum strength (**C**) and young’s modulus (**D**) of PVA and Az-Gel@PVA dressings. Error bars represent standard deviation for *n* = 3.

To investigate the influence of Az-Gel modification on the mechanical properties of the hydrogels, their tensile strength, the elongation at break and Young’s modulus were evaluated as shown in Figure 2. There was no significant difference between PVA with or without Az-Gel modification.

### 3.3. Cell Adhesion and Proliferation on the Az-Gel@PVA Dressing

**Figure 3:**
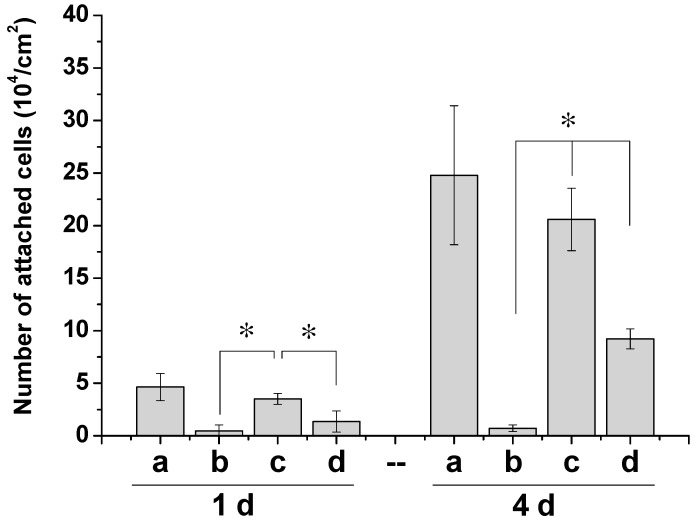
Cell counts of ADSCs on the different dressings after 1 and 4 d culture: tissue culture plate (TCP) (**a**), PVA (**b**), Az-Gel@PVA with and without UV irradiation (**c**,**d**). * *p* < 0.05, *n* = 4.

ADSCs were seeded in the grooves of PVA dressings and on the tissue culture plate (TCP) as control. Figure 3 showed that large number of cells adhered on the TCP control and Az-Gel@PVA dressings after 24 h, whereas only a small number of cells on PVA dressings without Az-Gel were modified. With prolonged incubation time, the differences between PVA dressings with and without Az-Gel modification became widened. Moreover, the number of attached cells on the Az-Gel@PVA dressings was close to that on the TCP. The effect of Az-Gel modification without UV-irradiation was also explored. Az-Gel solution with the concentration of 1 mg/mL was cast on the PVA dressings and directly washed without UV irradiation after the same persisted time. It was found that there were significant differences between the Az-Gel modified scaffolds with and without UV irradiation after 1 and 4 d culture. Obvious cell proliferation was observed on the modified scaffolds after UV irradiation compared to that without UV irradiation.

**Figure 4:**
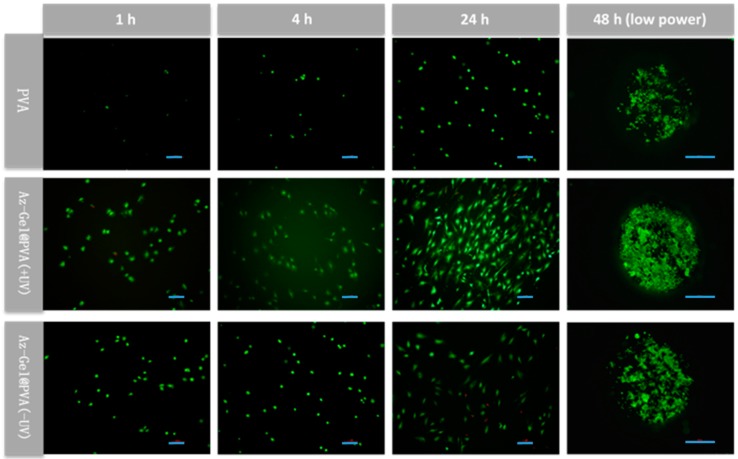
Fluorescence micrographs of live-dead staining of ADSCs on the different dressings after 1, 4, 24 and 48 h culture using Calcein-AM (Cal-AM) for live cells (green) and propidium iodide (PI) for dead ones(red). Scale bar lengths at 2, 4 and 24 h are 100 μm, while scale bar length at 48 h is 500 μm.

While cell counts are a good indication of cell survival, the cells on the gels were further observed by stained with PI and Cal-AM to get more information. Some cells attached to Az-Gel modified dressings at 1 h, whereas very few cells on the none-modified ones were observed (Figure 4). Afterwards, cells were observed spread on the Az-Gel modified dressings at 4 h. Only a few cells were found on the none-modified dressings. After 24 and 48 h culture, the cells were shown to be fully extended on all the dressings, but more extended cells were observed on the dressings with Az-Gel immobilization. The numbers of Cells attached on the Az-Gel modified dressings with and without UV irradiation were similar with the results of cell counts. Moreover, the low power observation showed that about all cells grew in the grooves, and few cells could be seen outside the grooves.

### 3.4. Protein Release from ADSCs-Seeded PVA Dressings

**Figure 5:**
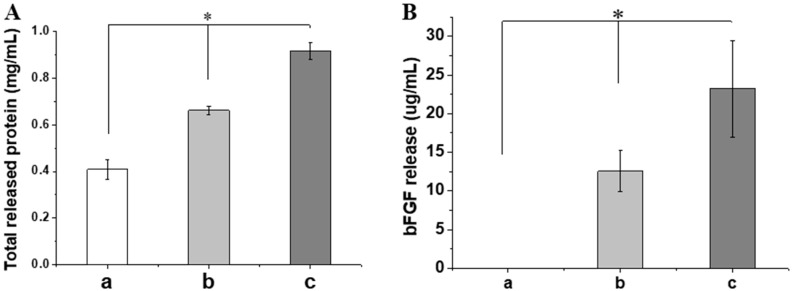
Total protein and basic fibroblast growth factor (bFGF) release from different dressings after incubated in serum-free medium for 48 h: Az-Gel@PVA dressings without ADSCs seeded (**a**), Az-Gel@PVA dressings with 3 × 10^5^ (**b**) and 6 × 10^5^ (**c**) cells/cm^2^ ADSCs seeded. * *p* < 0.05, *n* = 4.

Az-Gel@PVA dressings seeded with different densities of ADSCs (3 × 10^5^ and 6 × 10^5^ cells/cm^2^) were incubated in serum-free medium for 48 h and the released proteins from the patterned ADSCs/PVA dressings were quantitatively determined by BCA kit, to crudely reflect the released bioactive factors from dressings. Significant differences of protein release were found between Az-Gel@PVA dressings with and without seeded ADSCs. With more ADSCs seeded on the dressings, more released proteins were determined. To more specifically reflect the secreted active factors, bFGF was assessed via ELISA kit. As shown in Figure 5B, no bFGF was found in the dressings without ADSCs, but it could be detected in the ADSCs seeded groups. The trend of factor secretion in ADSCS seeded groups was similar to the total protein release.

### 3.5. NIH-3T3 Cells Proliferation

**Figure 6:**
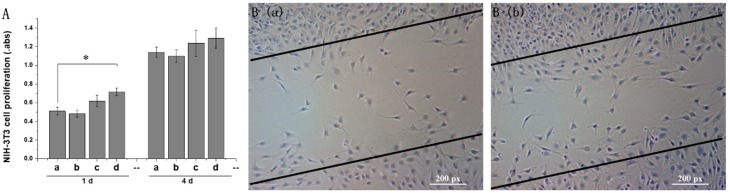
The ADSCs-seeded PVA dressings promoted NIH-3T3 cell proliferation and migration. (**A**) Cell Counting Kit-8 (CCK-8) test of NIH-3T3 proliferation covered with different dressings for 1 d and 4 d: control (a); Az-Gel@PVA dressing without ADSCs seeded (b); Az-Gel@PVA dressings with 3 × 10^5^ (b) and 6 × 10^5^ (c) cells/cm^2^ ADSCs seeded. * *p* < 0.05, *n* = 4. (**B**) The scratch closure in NIH-3T3 cells cultured for 24 h covered with Az-Gel@PVA dressings with (B(b)) and without (B(a)) ADSCs seeded. All scale bar lengths are 200 μm (px = μm in these images).

To determine the effect of ADSCs/Az-Gel@PVA dressings, NIH-3T3 cell, a mouse fibroblast cell line, was chosen as the model cell. NIH-3T3 cells were first seeded on a 12-well plate at an initial seeding density of 2 × 10^4^ cells/cm^2^. After overnight culture, the ADSCs/Az-Gel@PVA dressings were covered the plate to let the bioactive factors be secreted to the cells cultured in the plate. The proliferation of NIH-3T3 cell was shown in Figure 6. Compared to the control, PVA dressing without ADSCs seeded had little effect on the proliferation of NIH-3T3 cells. Dressings with different densities of ADSCs both performed high levels of OD values (Figure 6A). With prolonged incubation time, the ADSCs seeded dressings kept promoting the NIH-3T3 cell proliferation.

While cell proliferation is a good indication of tissue regeneration, the cell scratch experiment was further observed by stained with Trypan Blue to get more information. In this experiment, the scratch is assumed as the wound and the width of the scratch represents the degree of wound healing. In Figure 6B, two black lines were used to indicate the width of the scratch. It was found that the width of scratch covered with ADSCs seeded dressings (Figure 6B(b)) showed more narrow than that covered with PVA dressings (Figure 6B(a)). This result was consistent with the OD values of NIH-3T3 cells.

### 3.6. Wound Healing of Full Thickness Square Defects in Rats

**Figure 7:**
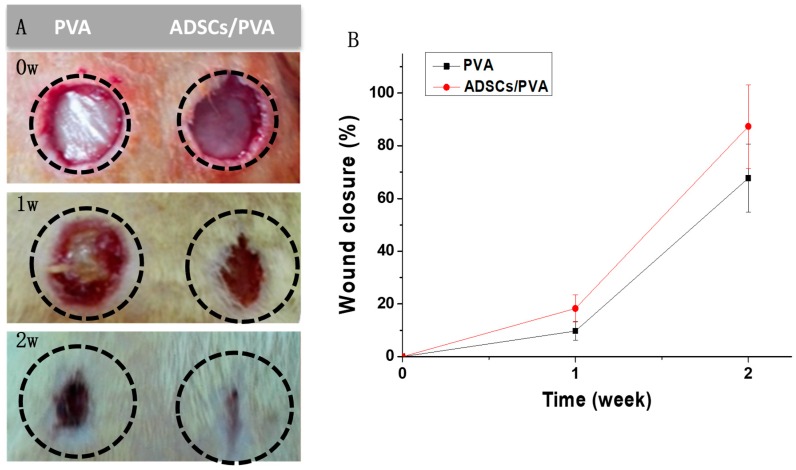
Photographs (**A**) and measurements (**B**) of wound healing of full thickness square defects in rats after different periods of time.

A preliminary attempt was made to repair the full thickness square defects in rats. It was observed that the wound closure after covered with different Az-Gel@PVA dressings. As shown in Figure 7, both of the photographs and measurements of wound closure indicated that the ADSCs/ADSCs/Az-Gel@PVA performed better effect of wound healing than the Az-Gel@PVA dressings without ADSCs seeded, which was similar with the in vitro study and further suggested the ADSCs/Az-Gel@PVA dressings could promote wound healing.

## 4. Discussion

In this study, we prepared PVA hydrogel dressings with a groove structure. The surface of the groove was photo-immobilized with Az-Gel to facilitate the adhesion and proliferation of stem cells. SEM images showed many filaments interlaced between the skeletal structures on the surface of the grooves modified with Az-Gel (Figure 1C), indicating that the surface of the groove was covered with Az-Gel. The repeated frozen-thawed treatment provided physical crosslinking of the PVA hydrogel with interpenetrating network structure by the intermolecular hydrogen bond formation [26]. It was reported that UV irradiation could crosslink pre-polymer solutions [27]. The mechanical properties of PVA dressings after surface modification were determined. But no significant differences of tensile strength, elongation at break and Young’s modulus were found between PVA with or without Az-Gel modification, which suggested that 20 s UV irradiation was too short to impact on the mechanical properties of PVA hydrogels. That is to say, the Az-Gel modification could not only improve PVA hydrogels surface properties, but also keep their mechanical properties. After SEM and mechanical characterization, ADSCs were seeded in the grooves to verify the cell adhesion and proliferation on the Az-Gel modified surface. As PVA hydrogel is a synthetic polymer without bioactive components incorporated to support cell attachment, cells do not attach to hydrogels made out of pure PVA [28]. After UV irradiation, azidophenyl groups of Az-Gel were photolyzed to generate highly reactive nitrenes that spontaneously formed covalent bonds with neighboring hydrocarbons on the polymer surface [29]. The Az-Gel covalent bond with PVA hydrocarbons and increased the bioactivity of the hydrogels, with approximately 100% seeding efficiency achieved. It was also found that more cells proliferated on the Az-Gel added PVA dressings without UV irradiation. But the cells that grew on its surface were much less than those on the modified surface via UV irradiation. It is also speculated that the improvement of the cell adhesion on Az-Gel added dressings without UV irradiation might be due to the residual Az-Gel in the hydrogel after washing. In this way, the ADSCs could adhere and proliferate on the surface of the dressing, whereas the bottom of the dressing could keep its non-adhesive property. The ADSCs seeded on the surface of the Az-Gel@PVA dressing could proliferated on it, which might secret bioactive factors to accelerate the wound healing. Meanwhile, the ADSCs seeded dressing would not adhere to the wound which would be convenient when changing new dressings. In order to roughly observe the cell distribution on the dressings, we also provided a low-power image of the ADSCs seeded dressings which showed about all cells grew in the grooves, and few cells could be seen outside the grooves. Even though the observation of cells on the dressings could not directly conform to no cells coming out of the dressings, it at least indicated that most of the cells grew in the groove. Further confirmation of whether ADSCs came out will be determined by markers in our future work.

As the purpose of the ADSCs/PVA dressing was to promote the wound healing via bioactive factors secreted by ADSCs. As known, ADSCs can secret many kinds of bioactive factors to accelerate wound healing, such as VEGF, bFGF and TGF-β [30]. The total protein release was determined to crudely reflect whether these ADSCs/Az-Gel@PVA dressings could secret bioactive factors to the wound. It was observed that the group of more ADSCs seeded Az-Gel@PVA dressings was determined more released protein, indicating the secreted bioactive factors could be released to the wound. The protein released from Az-Gel@PVA dressings was speculated the degraded and residual Az-Gel after washing. To further confirm our hypothesis, an ELISA assay was used to detect the amount of bFGF secretion. It was found that no bFGF was detect in the group without ADSCs seeded, which further indicated the protein in this group might be the degraded and residual Az-Gel. This could also show that with more ADSCs seeded, more bFGF was detected. So the total protein and bFGF release both indicated the ADSCs/Az-Gel@PVA dressings could release bioactive factors to the wound.

Wound healing is a complex process that goes through three phases: inflammation, proliferation, and tissue remodeling. Growth factors and cytokines, such as VEGF and bFGF, secreted at the wound site could decreased closure time, increased collagen deposition and granulation tissue formation [14]. In this study, in vitro and in vivo tests were both utilized to determine the effect of ADSCs/Az-Gel@PVA dressings to promote wound healing. In vitro cell proliferation showed that ADSCs/Az-Gel@PVA dressings performed promoting NIH-3T3 cells proliferation compared to the control and Az-Gel dressings without ADSCs seeded. It has been reported that ADSCs could express insulin-like growth factor-1 (IGF-1), VEGF-D and bFGF [31]. These factors are all of benefit to wound healing. The promoting of NIH-3T3 proliferation indicated that these factors could be secreted from ADSCs, penetrate through the dressing and accelerate wound healing. The cell scratch experiment showed that the width of scratch in the group of ADSCs/Az-Gel@PVA dressings was observed to be more narrow, which further conformed that the ADSCs/Az-Gel@PVA dressings played a role in the proliferation and migration of fibroblast cells. In vivo animal experiments were also carried out to further determine the effect of ADSCs/Az-Gel@PVA dressings. After 2 w of PVA dressings treatment, the group covered with ADSCs/Az-Gel@PVA dressings performed faster wound healing compared with that covered with dressings without seeded ADSCs. Among the bioactive factors secreted from ADSCs, it was reported that VEGF and bFGF could induce proliferation of endothelial cell and fibroblasts/keratinocytes, respectively [14]. TGF-β could promote cell migration to facilitate the wound closure [15]. Meanwhile, our group has also confirmed IGF-1 could promote the proliferation of many kinds of cells [24]. The in vivo test further indicated that the bioactive factors secreted from ADSCs could penetrate through the dressings to help wound repair. Above all, it was confirmed that the ADSCs/PVA dressings could secret and release some bioactive factors through hydrogels to the wound site. Among them, some factors, like VEGF, bFGF, TGF-β, IGF-1 and so on, might promote neovascularization, re-epithelialization and collagen deposition, and thus stimulate wound repair.

## 5. Conclusions

In this study, we prepared an ADSCs-seeded PVA dressing for wound healing. One side of the PVA dressings was modified with Az-Gel via UV irradiation, and thus ADSCs could adhere and proliferate on the PVA dressings and keep the other side of the dressings not adhering to the wound. Then some bioactive factors secreted from ADSCs could penetrate through the hydrogel to the wound site and promote wound healing without direct contact with the wound. It was speculated that these ADSCs/PVA dressings could not only promote wound healing, but also provide a new way for the safe application of stem cells. These stem cells seeded hydrogel dressings would be of great value for skin tissue engineering.
